# Comparing the pertussis antibody levels of healthy children immunized with four doses of DTap-IPV/Hib (Pentaxim) combination vaccine and DTaP vaccine in Quzhou, China

**DOI:** 10.3389/fimmu.2022.1055677

**Published:** 2023-01-06

**Authors:** Zhiying Yin, Canjie Zheng, Quanjun Fang, Tingcui Wen, Shuangqing Wang, Junji Li, Xiaoying Gong, Ziling Xiang

**Affiliations:** ^1^ Department of Immunity, Quzhou Center for Disease Control and Prevention, Quzhou, Zhejiang, China; ^2^ School of Public Health, Zhejiang Chinese Medical University, Hangzhou, Zhejiang, China

**Keywords:** pertussis IgG, Acellular pertussis vaccine, healthy children, purification methods, mathematic model

## Abstract

Despite the high coverage of pertussis vaccines in high-income countries, pertussis resurgence has been reported in recent years, and has stimulated interest in the effects of vaccines and vaccination strategies. Immunoglobulin G (IgG) antibodies against pertussis toxoid (PT), filamentous hemagglutinin (FHA), and pertactin (PRN) after immunization with four doses of co-purified or component vaccines were determined by enzyme-linked immunosorbent assay (ELISA). Serological data of PT-IgG geometric mean concentrations (GMCs) over time since vaccination were used to fit the mathematical models. A total of 953 children were included in this study; 590 participants received four doses of the component acellular vaccine and 363 participants received four doses of the co-purified acellular vaccine. The GMCs and the seropositivity rate of pertussis IgG were significantly influenced by the production methods, and the immunogenicity of the component acellular vaccine was superior to that of the co-purified acellular vaccine. The fitted mathematical models for the component acellular vaccine and the co-purified acellular vaccine were Y=91.20e^-0.039x^ and Y=37.71x^-0.493^, respectively. The initial GMCs of the component acellular vaccine was higher than that of the co-purified acellular vaccine, but both were similar at 72 months after immunization. Pertussis IgG levels waned over time after four doses of acellular pertussis vaccine, regardless of whether component or co-purified vaccine was used. The development and promotion of component acellular pertussis vaccines should be accelerated in China, and booster doses of pertussis vaccine in adolescents, adults, and pregnant women should be employed.

## Introduction

Pertussis (whooping cough) is a highly contagious respiratory infection that is caused by *Bordetella pertussis* (1). Although individuals in all age groups are susceptible, pertussis can be very serious and even fatal in infants. In the pre-vaccine era, pertussis epidemics peaked every 2 to 5 years, and up to 90% of household contacts and 50–80% of schoolroom contacts would be infected on exposure (2). Historical pertussis-related data in Quzhou indicates that, from 1957 to 1973, the average annual incidence was 138.69 per 100000 (40,983 pertussis cases) and the documented average annual mortality rate was 0.25 per 100000 (74 deaths) were recorded before the administration of pertussis vaccination. As pertussis is a vaccine-preventable infection, the introduction and widespread administration of the pertussis vaccine has dramatically decreased the incidence of pertussis. In China, the diphtheria–tetanus–pertussis combined vaccine (DTP), which comprised diphtheria and tetanus toxoids combined with a whole-cell pertussis vaccine (DTwP), was included in the Expanded Program of Immunization (EPI) in 1978. Subsequently, the average annual incidence decreased to 1.05 per 100000 and no death case was reported. As DTwP was produced by formalin inactivation of whole-cell pertussis organisms, a marked immune reaction was elicited and many children developed fever and 0.43% of vaccinees had febrile seizures (3). In 2008, the diphtheria, tetanus, and acellular pertussis combined vaccine (DTaP) was recommended to replace DTwP in Chinese children. The primary immunization program comprised vaccination at 3, 4, and 5 months of age, and a booster was administered at 18 months. However, no booster vaccination was available for susceptible populations including preschool children, adolescents, and pregnant women. The reported number of pertussis cases increased from 6658 in 2015 to 30,027 in 2019, and the actual number of cases was estimated to be 16.22 times higher in China (4), which has generated great concern from many experts. In England, a pertussis outbreak occurred in a junior school (ages 7–11 years) in March 2019 wherein the children had been offered a pertussis-containing booster vaccine at 40 months of age (5). In the past few years, countries with a high DTaP coverage have experienced a “pertussis resurgence” in the form of pertussis epidemics and/or local outbreaks (6, 7). This pertussis resurgence was attributed to the introduction of DTaP, which improved vaccination safety compared with DTwP (8). However, the waning of protection with DTaP appears similar to that of DTwP vaccines (9), and the main difference between DTaP and DTwP was in the pertussis component.

Owing to the pertussis resurgence, vaccine scientists are focusing on the pertussis infection and immunization, and is trying to develop more effective next-generation vaccines and vaccination strategies. Depending on the methods used for the production of acellular pertussis vaccine, the common DTaP vaccines that are used in China can be divided into two types: co-purified vaccines and component vaccines (10). Domestic manufacturers have primarily produced co-purified vaccines. As there was no separate purification of different antigen components during production, the proportion of antigens in the original solution varied according to the manufacturers and the production batches. A study (11) indicated that the co-purified pertussis vaccines that are produced in China contain not only the main antigen of purified pertussis toxoid (PT), but also include filamentous hemagglutinin (FHA), pertactin (PRN), fimbriae (FIM) 2 and 3, and other minor protein antigens. According to the Chinese pharmacopoeia, the dose of domestic co-purified pertussis vaccine was 0.5 ml per person and the titer of acellular pertussis antigens should not be less than 4.0 International Units (IU). For component vaccines, PT, FHA, PRN, and other antigens are extracted separately by column chromatography and then mixed in a precise proportion to ensure consistent quality between batches and to confer higher purity. However, the component vaccine technology is used in the production of only some imported DTaP vaccines (12). The component pertussis vaccine that is used in China comprises the diphtheria and tetanus toxoids and adsorbed acellular pertussis, inactivated poliovirus, and the *Haemophilus* b conjugate (tetanus toxoid conjugate) vaccine (DTaP-IPV/Hib) produced by Sanofi Pasteur, named as PENTAXIM, which comprises a 2-component acellular pertussis vaccine that contains 25 μg each of PT and FHA per 0.5 ml, and the immunization procedure is consistent with that of domestic DTaP (**Table 1**). Imported DTaP-IPV/Hib was used in Quzhou since 2011 although its use is limited by the high cost of the vaccine.

**Table 1:** Components and properties of DTaP-IPV/Hib and DTaP in each 0.5 mL dose.

Components	DTaP-IPV/Hib (PENTAXIM)	Domestic DTaP
Diphtheria toxoid	≥ 30 IU	≥ 30 IU
Tetanus toxoid	≥ 40 IU	≥ 40 IU
Acellular pertussis antigens	≥ 4.0 IU (including PT 25 μg and FHA 25 μg)	≥ 4.0 IU (including PT, FHA, PRN, FIM 2 and 3)
Inactivated polioviruses	type 1 (40 D-antigen units [DAU]); type 2 (8 DAU); type 3(32 DAU)	—
Haemophilus b conjugate (tetanus toxoid conjugate)	H. influenzae type b (10 μg); tetanus toxoid 18-30 μg	—
Accessories	Al(OH)3 et al.	Al(OH)3 1.0-1.5mg/ml, Thiomersal ≤ 0.1g/L et al.

Currently, China undertakes only passive surveillance for pertussis. Therefore, it is necessary to improve surveillance to determine the disease burden and to justify vaccination policies and recommendations, such as essential vaccination, boosters, and vaccination during pregnancy (13). Immunoglobulin G (IgG) antibodies against pertussis are indicators of the effectiveness of DTP vaccines in the vaccinated population. In this study, we compared anti-pertussis IgG levels between the co-purified vaccine (domestic DTaP) and the component vaccine (DTaP-IPV/Hib) by simulating the mathematical models of waning PT antibody following inoculation with an acellular pertussis vaccine in healthy children in Quzhou city, China. This study aimed to explore the attenuation regularity of serum antibodies against pertussis, this will promote the development of new vaccines, and provide a reference as a basis for adjusting immunization strategies to control pertussis transmission.

## Materials and methods

### Study design and participants

Quzhou is a prefecture-level city in the Zhejiang Province in eastern China that includes two districts and four counties. This cross-sectional study was conducted at two sites (Kecheng District and Jiangshan County) between September and December 2020 in Quzhou. Children who were vaccinated with four doses of DTaP-IPV/Hib vaccine in the Kecheng district and those vaccinated with four doses of domestic DTaP vaccine in Jiangshan county were recruited when their parents took them to the health centers for regular physical examination. All participants had no history of cough in the previous year and received DTP immunization information *via* the Zhejiang Provincial Immunization Information System (ZJIIS). Children with any of the following conditions were excluded: paroxysmal, spastic coughing sustained for more than 2 weeks within the last year, febrile disease, respiratory disease, immunodeficiency disorder, thrombocytopenia, or any other bleeding disorder.

Eligible participants were divided into seven age groups: <2, 2, 3, 4, 5, 6, and 7–9 years. The sample size for each group was based on the following formula: n=*Z_α_^2^P*(1-*P*)/*d^2^*, *where d* is the tolerance error, *P* is the seropositivity rate of pertussis IgG, *α*=0.05, *d*=9%. The seropositivity rate after four doses of the acellular pertussis vaccine was estimated as 92%, and the minimum sample size of each group was 36, the recruitment for each age group ranged from 60 to 80. A total of 420 children in the DTaP group were recruited, and 55 of whom received only three doses of domestic DTaP vaccine, 2 of whom had no history of domestic DTaP vaccine. And 596 children in the DTaP-IPV/Hib group were recruited, 6 of whom received only three doses of DTaP-IPV/Hib vaccine. Finally, 590 eligible children in the DTaP-IPV/Hib group and 363 in the domestic DTaP group were included in this study.

### Specimen collection and laboratory testing

Blood samples (2 mL venous blood) were collected from each participant, and the serum was separated and placed in a centrifuge tube with a screw cap. The serum was placed in a cryopreservation box and stored at −80°C in a freezer. IgG antibodies against pertussis were measured quantitatively using an in-house enzyme-linked immunosorbent assay (ELISA). Briefly, 96-well ELISA plates were coated with purified PT, FHA, or PRN, and blocked with skim milk solution. WHO Human International Standard for Pertussis Antiserum (NIBSC 06/140) was used for this assay. The antibody concentration was generated using a standard curve obtained from point-to-point plotting (linear/linear) of the optical density values. The ELISA kit that used in our study is an in-house kit developed by Whuan Institute of Biological Products CO., LTD which provide reference to China National Institute for Food and Drug Control (14). Test results for pertussis antibodies (anti-PT, anti-FHA, or anti-PRN) were considered positive if their antibody concentration was ≥20 International Units per mL (IU/mL); otherwise, the test result was considered negative. Pertussis IgG antibody positivity indicates past pertussis infection, successful vaccination, or immunoglobulin-recipient status.

### Curve fitting

Basic principle (15): The decay trend of vaccine antibodies mostly presents an exponential curve or power curve, in the General Model Ŷ=k+a×exp(bX) or Ŷ=k+aX^b^, respectively. The exponential curve occurs when the independent variable Xi changes according to the series of equal differences, and the strain Yi changes according to the series of equal differences. The power curve occurs when the independent variable Xi changes in an equal series, and Yi also changes in an equal series. Accordingly, it is possible to choose whether the fitted model is an exponential or a power curve, and the mathematical model of the measured data is obtained.

Fitting process: The time-independent variables Xi (1, 12, 24, 36, 48, 60, and 72 months) and the PT-IgG-dependent variable Yi were established using SPSS for Windows (version 16.0, SPSS Inc., USA). After selecting “Analysis”→”Regression”→”Curve estimation,” we selected Yi and Xi into the list of dependent and independent variables, respectively, and selected “Exponential,” “Power”, and remove “Linear” commands in the models. Next, we selected the “Display ANOVA table” at the bottom and clicked OK. On comparing the R value and the determining coefficient R^2^ of the “Exponential” model and the “Power” model, a bigger R^2^ indicated better model fitting.

### Statistical analysis

Data were collected using Microsoft Office Excel (version 2007). The geometric mean concentration (GMC), 95% confidence interval (95% CI), and seropositivity rate (SPR) were calculated. The comparison of GMC was performed using two independent variable *t*-tests, and the rates was compared using the chi-square test. Statistical analyses were performed using SPSS for Windows (version 16.0; SPSS Inc., USA) and GraphPad Prism 5 statistical packages. Statistical significance was set at a two-sided *p*-value <0.05.

## Results

### Sociodemographic characteristics

A total of 953 children (512 boys and 411 girls) were included in the study, and 590 participants received with four doses of DTaP-IPV/Hib were assigned to the DTaP-IPV/Hib group whereas 363 participants received with four doses of domestic DTaP were assigned to the DTaP group. According to the difference between the sampling time and the time of the fourth dose, the 953 participants were stratified into seven age groups: 1, 12, 24, 36, 48, 60, and 72−96 months. The sample sizes among 7 various age groups were 36, 84, 121, 112, 135, 53, and 49 in the DTaP-IPV/Hib group and 45, 64, 58, 49, 72, 29, and 46, respectively, in the DTaP group. The sex ratio and mean age among 7 various age groups did not differ significantly between the DTaP-IPV/Hib and DTaP groups (*p* > 0.05; **Table 2**).

**Table 2:** Comparison of basic characteristics between the two study groups.

Time since last dose (months)	Sex (male/female)				Age (years, 95% CI)			
	DTaP-IPV/Hib	DTaP	*X* ^2^	*P*	DTaP-IPV/Hib	DTaP	*t*	*p*
1	17/19	28/17	1.82	0.17	2.17 (2.08–2.25)	2.18 (2.07–2.28)	0.16	0.87
12	44/40	34/30	0.01	0.93	3.17 (3.10–3.24)	3.22 (3.09–3.36)	0.66	0.51
24	64/57	35/23	0.88	0.35	4.23 (4.16–4.30)	4.14 (4.00–4.28)	1.20	0.23
36	61/51	27/22	0.01	0.94	5.31 (5.23–5.40)	5.24 (5.12–5.36)	1.07	0.29
48	65/70	42/30	1.95	0.16	6.15 (6.09–6.21)	6.13 (6.06–6.20)	0.45	0.65
60	25/28	18/11	1.67	0.20	7.07 (6.97–7.17)	6.99 (6.86–7.13)	0.90	0.37
72–96	25/24	27/19	0.56	0.45	8.21 (8.10–8.32)	8.28 (8.17–8.40)	0.86	0.38

### GMCs of pertussis IgG

The GMCs of PT-IgG at 1, 12, 24, 36, 48, 60, and 72-96 months after four doses of DTaP-IPV/Hib were 98.91, 63.13, 45.46, 13.91, 10.38, 8.88, and 8.17 IU/mL, respectively, which were all higher than those quantified at the same time after four doses of domestic DTaP. The GMCs of PT-IgG after immunization in the two groups waned over time; however, the GMCs at 72–96 months after the fourth dose in the DTaP group showed a slight increase. Furthermore, the study showed that the GMCs of FHA-IgG among the seven age groups in the DTaP-IPV/Hib group were higher than those in the DTaP group. The GMCs of FHA-IgG after 12 months in the DTaP-IPV/Hib group decreased slowly, but in the DTaP group, the tendency was consistent with that of PT-IgG, which waned after immunization but increased at 72–96 months. The GMCs of PRN-IgG in the DTaP-IPV/Hib group ranged from 2.49 to 3.70 IU/mL, which showed irregularity among the seven age groups. The lowest GMCs of PRN-IgG was 15.19 IU/mL in the 24 months group whereas the highest was 49.77 IU/mL in the 1 month group, which showed a “U” trend of initial decline and subsequent increase in the DTaP group (**Table 3**).

**Table 3:** The GMCs of pertussis IgG between the two groups.

Time since last dose (months)	DTaP-IPV/Hib (n=590)		DTaP (n=363)		t*	*p*	
	No. of subjects	GMC (IU/mL, 95% CI)	No. of subjects	GMC (IU/mL, 95% CI)			
PT-IgG							
1	36	98.91 (68.25–143.30)	45	33.14 (23.19–47.38)	4.26	<0.001
12	84	63.13 (53.85–74.01)	64	13.32 (10.16–17.47)	9.90	<0.001
24	121	45.46 (40.10–51.53)	58	11.51 (9.05–14.63)	10.13	<0.001
36	112	13.91 (11.97–16.16)	49	5.83 (4.24–8.02)	5.59	<0.001
48	135	10.38 (9.04–11.91)	72	4.47 (3.74–5.34)	7.27	<0.001
60	53	8.88 (7.40–10.66)	29	4.26 (2.63–6.89)	2.92	0.006
72–96	49	8.17 (6.34–10.54)	46	4.87 (3.81–6.22)	2.95	0.004
FHA-IgG							
1	36	149.30 (105.90–210.30)	45	66.21 (44.74–98.00)	3.07	0.003
12	84	58.32 (48.78–69.74)	64	25.69 (19.42–33.97)	4.93	<0.001
24	121	53.46 (44.45–64.28)	58	17.81 (13.17–24.07)	6.47	<0.001
36	112	48.43 (39.40–59.52)	49	10.27 (6.35–16.60)	5.95	<0.001
48	135	48.22 (40.93–56.80)	72	8.50 (6.55–11.03)	11.65	<0.001
60	53	34.76 (27.51–43.92)	29	7.61 (4.76–12.18)	5.91	<0.001
72–96	49	32.30 (26.18–39.85)	46	11.68 (8.37–16.29)	5.21	<0.001
PRN-IgG							
1	36	3.19 (2.56–3.98)	45	49.77 (32.69–75.78)	10.85	<0.001
12	84	3.36 (2.97–3.81)	64	20.78 (16.20–26.67)	13.13	<0.001
24	121	3.70 (3.33–4.13)	58	15.19 (11.11–20.78)	10.54	<0.001
36	112	3.08 (2.68–3.54)	49	17.01 (10.87–26.62)	9.43	<0.001
48	135	2.49 (2.24–2.77)	72	16.45 (11.30–23.95)	12.14	<0.001
60	53	2.38 (1.94–2.92)	29	22.46 (13.54–37.23)	8.40	<0.001
72–96	49	2.88 (2.37–3.50)	46	20.16 (13.35–30.46)	8.77	<0.001

*the value of t test in each age group between the DTaP-IPV/Hib group and the DTaP group.

### Seropositivity rate of pertussis IgG

**Figure 1:**
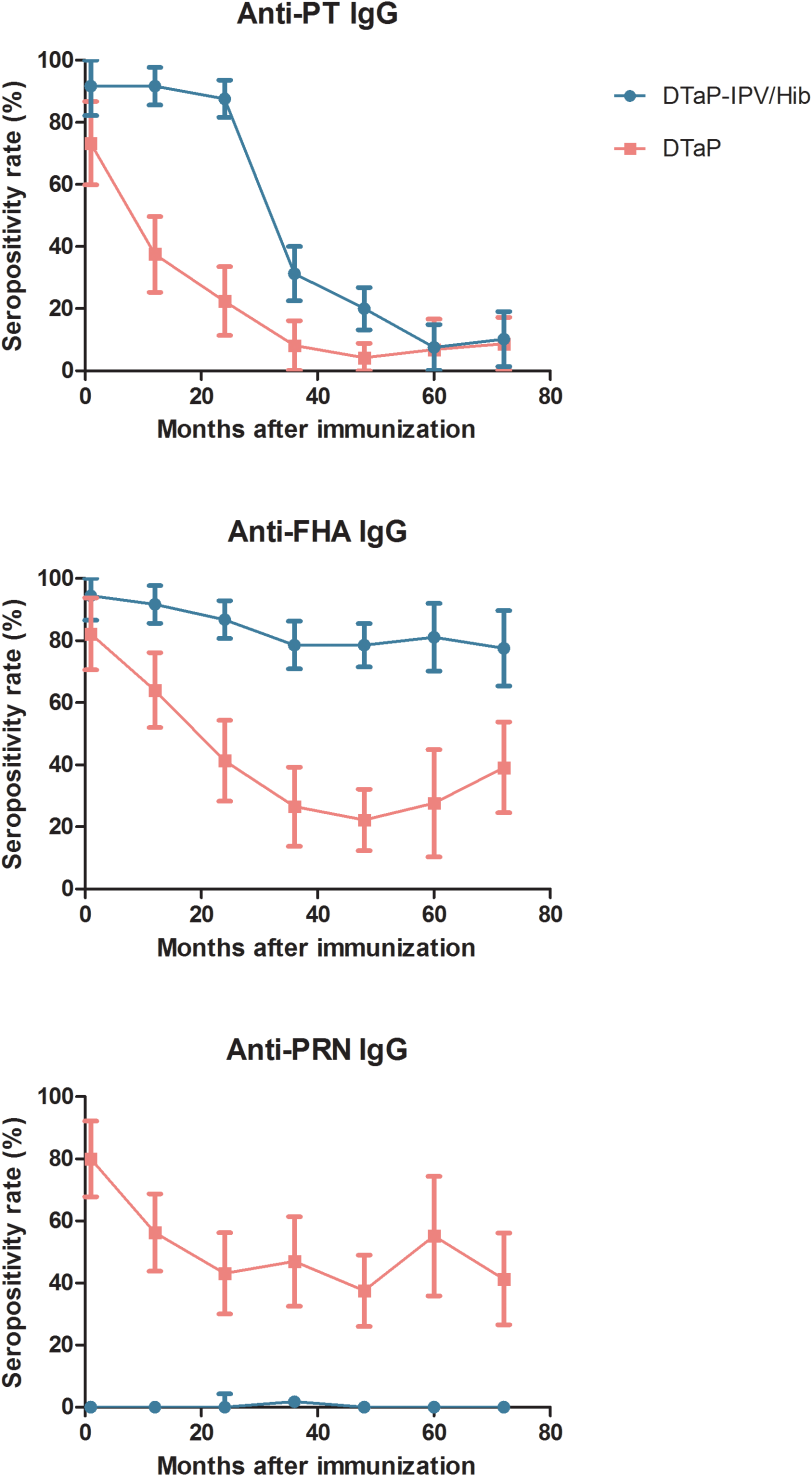
Seropositivity rate of pertussis IgG between the two groups.

The seropositivity rates of anti-PT IgG at 1, 12, 24, 36, 48, 60, and 72–96 months in the DTaP-IPV/Hib group were 91.67% (33/36), 91.67% (77/84), 87.60% (106/121), 31.25% (35/112), 20% (27/135), 7.55% (4/53), and 10.20% (5/49), respectively, which initially declined slowly and then rapidly before plateauing at 60 months after immunization. The seropositivity rate of anti-PT IgG in the DTaP group was 73.33% (33/45), 37.50% (24/64), 22.41% (13/58), 8.16% (4/49), 4.17% (3/72), 6.90% (2/29), and 8.70% (4/46), which were lower in the 60 months following immunization than those at the same time in the DTaP-IPV/Hib group; thereafter, the seropositivity rate was similar in the two groups. The seropositivity rate of anti-FHA IgG after four doses of DTaP-IPV/Hib ranged from 77.55% (38/49) in the 72–96 months group to 94.44% (34/36) in the 1 month group, which showed a slow downward trend. However, in the DTaP group, the rate of anti-FHA IgG ranged from 22.22% (16/72) in the 48-months group to 82.22% (37/45) in the 1 month group, which was considerably lower than that in the DTaP-IPV/Hib group. The seropositivity rate of anti-PRN IgG ranged from 37.50% (27/72) to 80.00% (36/45) in the DTaP group, and the rates among the seven age groups were close to 0 in the DTaP-IPV/Hib group (**Figure 1**).

### Mathematical models of waning anti-PT IgG

**Figure 2:**
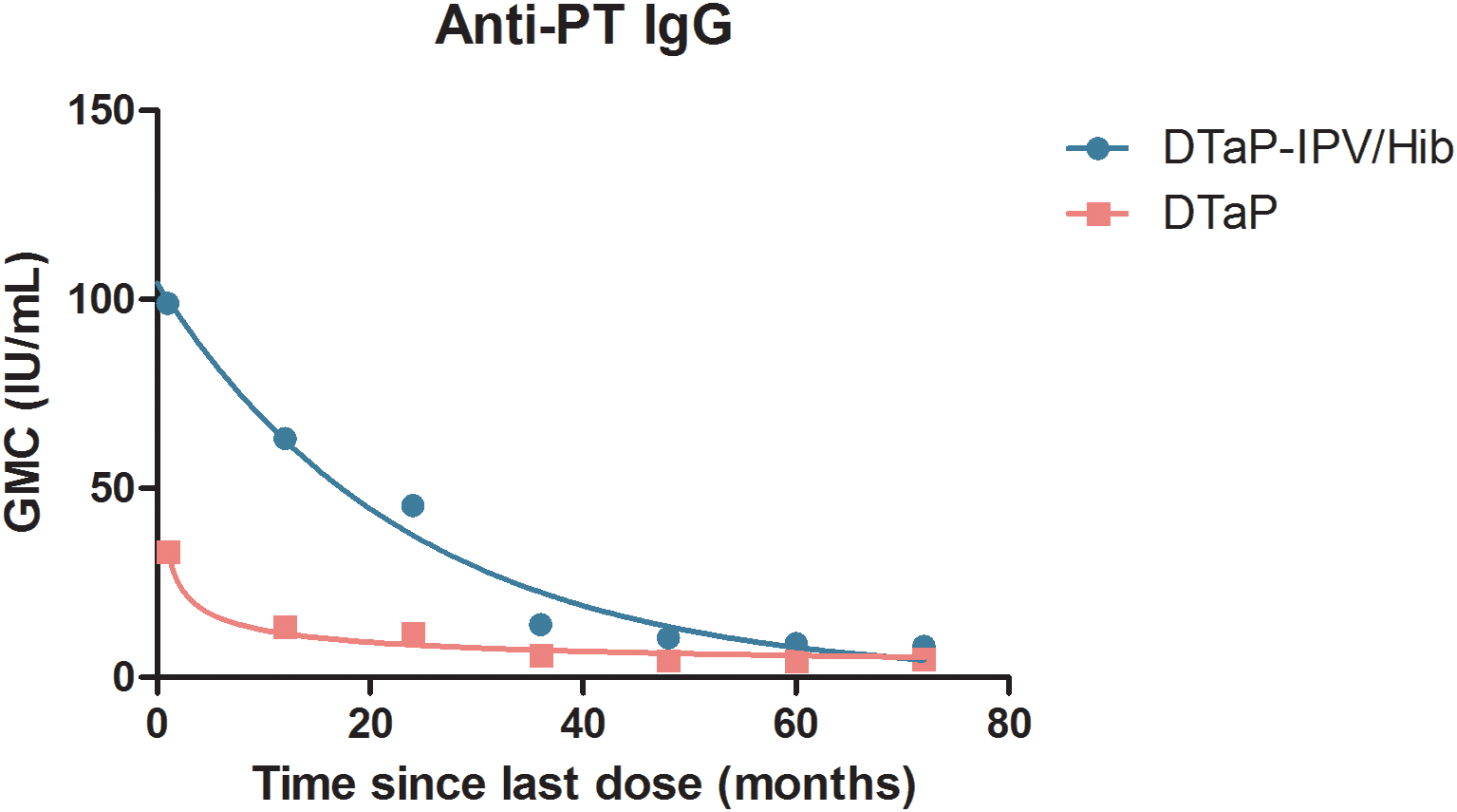
The curve fit of waning anti-PT IgG for the two groups.

On comparing the determining coefficient R^2^, the exponential curve was better than the power curve for anti-PT IgG in the DTaP-IPV/Hib group, conversely, the power curve was superior to the exponential curve for anti-PT IgG in the DTaP group (**Table 4**). Therefore, the fitted mathematical model of waning anti-PT IgG for the DTaP-IPV/Hib group was Y=91.20e^-0.039x^ (*R^2^* = 0.914, *F* = 53.32, *P* = 0.001), and the fitted model for the DTaP group was Y=37.71x^-0.493^ (*R^2^* = 0.918, *F* = 56.00, *P* = 0.001). **Figure 2** shows the waning trend of anti-PT IgG in the two groups, the initial GMCs after immunization of the DTaP-IPV/Hib group were higher than those of the DTaP group, whereas the GMCs of the DTaP-IPV/Hib group decreased rapidly, and the GMCs of the two groups were similar at 72 months.

**Table 4:** The model summary and parametric estimates of anti-PT IgG for the two groups.

Equation	Model Summary			Parametric Estimates	
	R Square	F	P	Constant	b1
DTaP-IPV/Hib					
Power	0.771	16.88	0.009	149.12	−0.615
Exponential	0.914	53.32	0.001	91.20	−0.039
DTaP					
Power	0.918	56.00	0.001	37.71	−0.493
Exponential	0.820	22.79	0.005	21.95	−0.027

## Discussion

Pertussis resurgence has become an international public health concern and has a complex multifactorial causation, including the fact that natural infections produced limited immunity, the definitions of surveillance cases varied country-wise, occurrence of atypical symptoms, limited laboratory diagnostic ability, vaccination coverage, attenuated vaccine protection, and so on. Our study mainly focused on the impact of the vaccine on pertussis resurgence. Despite the DTaP coverage was over 90% in China, the number of reported pertussis cases has steadily increased in recent years. A large pertussis outbreak occurred in a primary school with a high vaccination coverage in Tianjin (16). Therefore, two vaccine-related issues must be considered: the composition of the vaccine and the immunization schedule.

Evidence suggests that a pertussis vaccination confers short-term disease protection, although this protection wanes rapidly with acellular pertussis vaccines (17). Our research showed that the GMCs and the seropositivity rates of PT-IgG and FHA-IgG waned over time even after four doses of DTaP-IPV/Hib, but the levels were all higher than those quantified at the same timepoints in the DTaP group. The main difference of acellular pertussis antigens between DTaP-IPV/Hib and DTaP was the production method; DTaP-IPV/Hib is a component vaccine, whereas DTaP is a co-purified vaccine. The number of bioactive proteins in component acellular pertussis vaccines is generally higher than that in co-purified vaccines (18). Furthermore, co-purified acellular vaccines that are used in China induce lower levels of anti-pertussis toxin IgG than separately purified acellular vaccines (19), and previous studies suggested that separately purified acellular pertussis vaccines were used in the national immunization program. On the other hand, the fact that DTaP-IPV/Hib (PENTAXIM) has two more pathogenic “valencies” (IPV, HIB) cannot be ignored, whether this should affect the antibody levels remains to be further studied. The GMCs and the seropositivity rate of PT-IgG and FHA-IgG increased at 72–96 months after the last dose in the DTaP group, possibly because children born between 2010 and 2012 had natural infection that caused by low immunity; moreover, it is possible that the information system was not established very well before 2012, and some children who vaccinated with DTwP were incorrectly recorded as DTaP. Thus, although the GMCs of PT-IgG and FHA-IgG after immunization with four doses of DTaP-IPV/Hib were higher than those of DTaP under the same conditions, the imported DTaP-IPV/Hib that was used in China was a 2-component acellular pertussis vaccine that contained only PT and FHA, lack of PRN. The GMCs of PRN-IgG in the DTaP group showed up-down-up, which may be related to the unbalance of pertussis components in the co-purified process. PRN is a common acellular vaccine antigen, and the number of PRN-deficient *B. pertussis* isolates has progressively increased in countries where acellular pertussis vaccines are used, including in France, Italy, Japan, and the United States (20). Moreover, the proportion of PRN-negative *B. pertussis* that was isolated in any country positively correlated with the time that had elapsed since the transition from whole-cell to acellular pertussis vaccine (21). Vaccine-induced immunity exerts selection pressure on *B. pertussis*, which leads to antigenic drift and loss of expression of vaccine antigens (22). Thus, PRN-deficient strains would provide useful information to facilitate the current search for new protective antigens and generate broader lessons for the design of improved subunit vaccines (23). Therefore, it is important for China to develop separately purified acellular pertussis vaccines that contain the PT, FHA, and PRN components and to introduce them into the EPI to reduce the prevalence and spread of pertussis.

Undervaccination with the pertussis vaccine leads to a higher risk of pertussis. Short delays in vaccine administration were considered less important if an age-appropriate number of doses was administered; however, delayed vaccination was not recommended. Even if there is some delay, it is important for children to receive all doses of the pertussis vaccine (24). In China, the current vaccination schedule of acellular pertussis vaccine includes three primary doses at a minimum starting age of 3, 4, and 5 months and a booster dose at a minimum starting age of 18 months, which is consistent with the schedule of acellular pertussis vaccination in France. Paireau and colleagues (25) compared the subsequent effect of the old and new French schedules, which includes two primary doses at 2 and 4 months of age and the first booster at 11 months of age and has only been implemented from April to May 2013; the authors reported that the new schedule increased the risk for pertussis and decreased anti-pertussis toxin IgG levels. Therefore, it is important to access enough doses. The mathematical models of waning anti-PT IgG showed that the initial GMCs after immunization of the DTaP-IPV/Hib group were higher than those of the DTaP group; however, the GMCs of the two groups were basically the same at 72 months after immunization. Our data suggest that the current schedule offour doses of acellular pertussis vaccine is inadequate in China, and it would be necessity to modify the pertussis vaccination strategy to administer booster doses for preschool children, adolescents, and pregnant women.

The waning immunity among the cohort that received the acellular pertussis vaccine was thought to drive the pertussis resurgence in the United States (26). The effectiveness of administering four doses of the pertussis vaccine during infancy decreases with the time since the fourth dose administration. However, this regimen does not protect school-aged children against pertussis (27). Consistent trends of decreasing pertussis vaccine effectiveness with increasing time since the last vaccination across three Canadian provinces indicated the need for immunization schedules and vaccine development to optimize protection for all individuals, especially adolescents and young adults who carry the greatest risk of infection (28). Students who had received the last dose of pertussis vaccine more than 4 years earlier were three times more likely to become ill than those with a booster dose less than 4 years earlier (16). Adding a booster vaccination at the age of 5–6 years may reduce the incidence and mortality of pertussis in young infants in China. The first dose of vaccination for the child is at 3 months, leading to a longer unprotected window for younger infants. Maternal immunization strategy with adult-formulated tetanus–diphtheria–pertussis vaccine (Tdap) has already been included in some countries. Research in Costa Rica showed that the introduction of booster doses of DTP and Tdap postpartum immunization has a greater effect on decreasing hospitalizations and deaths due to pertussis (29). Research in Quzhou showed that the GMC of PT-IgG antibody in umbilical cord blood was 19.12 IU/ml, and only 32.26% of newborns had a PT-IgG antibody concentration ≥30 IU/mL (30), which showed limited levels of pertussis antibodies in healthy children born from mothers. The current pertussis immunization targets in China do not include pregnant women, which results in infants receiving little pertussis antibody from the mother. Therefore, developing domestic Tdap, introducing imported Tdap, and implementing booster doses for adolescents or pregnant women are important for preventing the transmission of pertussis in China.

Our study had several limitations. First, no uniform pertussis serum antibody detection reagents and criteria can be used in China, which may lead to differences in the seropositivity rates in different studies, and it is impossible to determine recent infection or successful vaccination in individuals with high pertussis IgG antibody levels. Second, the study was cross-sectional, and the conclusions were not as persuasive as those of a cohort study; therefore, cohort research needs to be conducted to validate our findings. The participants were recruited according to their age, which led to different sample sizes after immunization among the various age groups. Third, there are more differences between DTaP-IPV/Hib (PENTAXIM) and domestic DTap except the purification method, the effects of different components on antibody levels need to be further studied.

In conclusion, the GMCs and seropositivity rate of pertussis IgG with DTaP-IPV/Hib (PENTAXIM) vaccine were significantly higher than that of domestic DTaP, which may be influenced by the production methods, and the immunogenicity of the component acellular pertussis vaccine was superior to that of the co-purified acellular vaccine. The pertussis IgG waned over time after four doses of acellular pertussis vaccine, either with a component or co-purified vaccine, and ultimately failed to protect individuals against infection. In China, development and promotion of component acellular pertussis vaccine should be accelerated, and booster doses of the pertussis vaccine in adolescents, adults, and pregnant women should be employed.

## Data availability statement

The raw data supporting the conclusions of this article will be made available by the authors, without undue reservation.

## Ethics statement

This study was approved by the Ethics Committee of Zhejiang Provincial Centers for Disease Control and Prevention (2020014) and the Ethics Committee of Quzhou Centers for Disease Control and Prevention (2019003). All participants were given a brief oral description of the aims of the study, and written informed consent was obtained from the legal guardians of the children before the enrolment.

## Author contributions

ZY conceived and wrote the manuscript. CZ, XG, QF and JL collected and organized the data. TW and SW analyzed the data. ZX revised and edited the manuscript. All authors contributed to the article and approved the submitted version.
